# Evaluation of the Cataract Surgery 2018 Survey in Terms of Achieving Refractive Cataract Surgery Targets

**DOI:** 10.4274/tjo.galenos.2020.46020

**Published:** 2021-02-25

**Authors:** İzzet Can, Tamer Takmaz, Akif Özdamar, Ümit Kamış, Yonca Aydın Akova, Osman Şevki Arslan, Mehmet Baykara, Kazım Devranoğlu, Üzeyir Günenç, Fatih Mehmet Mutlu, Altan Atakan Özcan, Emrullah Taşındı

**Affiliations:** 1 Private Practice, Ankara, Turkey; 2 Ankara City Hospital, Clinic of Ophthalmology, Ankara, Turkey; 3 İstanbul University-Cerrahpaşa, Cerrahpaşa Faculty of Medicine, Department of Ophthalmology, İstanbul, Turkey; 4 Konya Dünyagöz Hospital, Konya, Turkey; 5 Bayındır Hospital, Ankara, Turkey; 6 Uludağ University Faculty of Medicine, Department of Ophthalmology, Bursa, Turkey; 7 Private Practice, İstanbul, Turkey; 8 Dokuz Eylül University Faculty of Medicine, Department of Ophthalmology, İzmir, Turkey; 9 University of Health Sciences, Gülhane Faculty of Medicine, Ankara, Turkey; 10 Çukurova University Faculty of Medicine, Department of Ophthalmology, Adana, Turkey; 11 Okan University Faculty of Medicine, Department of Ophthalmology, İstanbul, Turkey

**Keywords:** Refractive cataract surgery, cataract survey, phacoemulsification

## Abstract

**Objectives:**

The aim of this study was to show at what rate the technological equipment used in cataract surgery by Turkish ophthalmologists and their knowledge are reflected in practice and how up to date they are.

**Materials and Methods:**

A questionnaire conducted using SurveyMonkey was used to evaluate the answers to 17 questions from 823 members of the Turkish Ophthalmological Association. Results were evaluated in subgroups according to the participants’ age, occupational status, institutions, and whether they conducted relevant academic activities, and the data were compared as inadequate, standard, and contemporary approaches according to the determined criteria.

**Results:**

Optical biometry devices were used at rates of 77.7% and 67.3% for intraocular lens (IOL) power calculations and keratometric measurements in preparation for cataract surgery, respectively. For IOL power calculation, third-generation formulas, especially the SRK-T, were used most commonly (46.2%), followed by second-generation formulas (21.9%), and fourth/fifth-generation formulas and multiple evaluations for different axial lengths (31.9%). The most common incision size was 2.8 mm (51.6%), while the percentage of 2.2 mm and shorter incisions considered to be neutral in terms of surgically induced astigmatism was 18.8%. When selecting incision location, approaches to reduce corneal astigmatism were reported by 28.9%, neutral approaches by 26.2%, and insensitive approaches by 44.9%. Additionally, 55.6% of participants never implanted toric IOLs and 50.7% did not use presbyopia-correcting IOLs. The proportion of surgeons who have experience with femtosecond laser-assisted cataract surgery was 10.3% and the rate of intracameral antibiotic injection at the end of the operation was 89.4%.

**Conclusion:**

It was seen that Turkish cataract surgeons were able to use high technology for surgical preparation and surgery at high rates, but this was not reflected in practice at same rate in terms of achieving contemporary standards of refractive cataract surgery.

## Introduction

Modern cataract surgery is no longer an approach that provides visual rehabilitation by removing the opacified lens only, but has become a refractive surgery that aims to eliminate the patients’ other visual problems as well. In other words, cataract surgery has come to a point where greater importance is placed on correcting or reducing patients’ existing aberrations, especially astigmatism, eliminating refractive errors and presbyopic complaints, and doing all of them with attention to safety as well as functionality. In this environment of increasing patient expectations and demands, the ability to accurately predict postoperative outcomes has become dependent on meticulous preoperative preparation using advanced technology.^1,2^ Other important factors when evaluating contemporary or current approaches are the extent to which surgeons consider surgically induced astigmatism (SIA) during surgery, use premium intraocular lenses (IOL), and take measures for endophthalmitis prophylaxis.

The present study aimed to determine what proportion of Turkish ophthalmologists meet the above-mentioned criteria in their approaches to cataract surgery, or in other words, their level of practice development.

## Materials and Methods

Using the SurveyMonkey data platform (http://tr.surveymonkey.com), an online questionnaire consisting of 33 questions was sent to 4501 members of the Turkish Ophthalmological Association (TOA) in April 2018, and responses from 823 of those ophthalmologists were collected. The questions included in the questionnaire were grouped under 7 main headings: 1) Cataract surgery preparation, 2) Cataract surgery techniques used, 3) Approach to femtosecond laser-assisted cataract surgery (FLACS), 4) Approach to astigmatic patients and toric IOL implantation, 5) Approach to presbyopia correction in cataract surgery, 6) Reasons for not using techniques and technologies related to refractive cataract surgery, and 7) Questions about other refractive surgeries. Of the 33 questions asked in this questionnaire, 17 were considered relevant to the aim of this study and were included in the evaluation.

The respondents were classified into 4 different subgroupings based on the following variables: 1) Professional status (resident; specialist; faculty member), 2) Employing institution (independent/private practice; private medical center/hospital or foundation hospital; secondary public hospital; tertiary public hospital [education hospital]; public university; private/foundation university), 3) Academic activities related to cataract surgery (whether an active member of TOA Society of Cataract and Refractive Surgery [SCRS]), and 4) Age (20-30; 31-40; 41-50; 51-60; and >60 years).

### Statistical Analysis

These subgroups were compared statistically using IBM SPSS Statistics Version 21 package program (IBM Corp, Armonk, NY). Non-parametric, categorical variables were analyzed using Pearson’s chi-square test. In addition, z-tests were used to compare the reported preferences within the groups. Data with p-values less than 0.05 were accepted as statistically significant.

When the participants’ professional status was examined, 78 of the 823 participants were residents (9.5%), 487 were specialists (59.2%), and 258 were faculty members (31.3%). Thirty-eight respondents (4.6%) were independent practitioners, 247 (30.0%) worked at a private medical center, private hospital, or foundation hospital, 137 (16.6%) at secondary pubilc hospitals, 240 (29.1%) at tertiary public hospitals, 148 (17.9%) at public universities, and 52 (6.3%) at private/foundation universities. One hundred forty (17.0%) of the participants were active members of TOA-SCRS and 683 (83.0%) were not. The age distribution of the respondents was as follows: 20-30 years: 90 (10.9%); 31-40 years: 310 (37.7%); 41-50 years: 196 (23.8%); 51-60 years: 167 (20.3%), and over 60 years: 60 (7.3%). The overall mean age was 42.53 years.

The monthly volume of cataract surgeries performed by the participants is shown in Table 1.

After documenting the current situation, we aimed to conduct a secondary analysis to determine the level of development in the field of cataract surgery in Turkey in terms of achieving refractive cataract surgery targets. To do this, we attempted to investigate the extent to which Turkish ophthalmologists apply their knowledge of and experience with the technological equipment they have access to and use for refractive cataract surgery and to what extent the surgical techniques and approaches they use serve the purpose.

For this study, the responses obtained for 5 of the 17 questions considered relevant to the aim of this study were compared between the 4 subgroupings defined above. For each question, the answers were grouped as “inadequate” approaches, “standard” approaches, and “contemporary” approaches (Table 2). Classification of responses as inadequate, standard, or contemporary was based on the current literature data ^3,4,5^ and the results of recent large-scale international surveys.^6,7^

## Results

### Available Technology

Responses to three questions were used to collect information about the technological equipment that the respondents had at their disposal.

The first question was “What device and technique do you most use for IOL power measurement? (More than one option can be marked)”, to which 77.7% of participants said an optical biometry (IOLMaster, Carl Zeiss Meditec, Germany; Lenstar, LS900, Haag-Streit AG, Switzerland; AL-Scan, Nidek Co., Ltd, Japan; Aladdin, Topcon, Japan), 35.3% said contact ultrasound (A-scan), and 8.5% said immersion ultrasound (A-scan).

The second question was asked to learn about the equipment owned (“What is the most common keratometry method you use in routine IOL calculation?”), and responses were manual keratometry (1.2%), autokeratometry (29.6%), optical biometry (e.g., IOLMaster, Lenstar, AL-Scan, Aladdin; 67.5%), and corneal topography/corneal analyzer (1.7%).

When asked “What are your thoughts on the future of FLACS?”, 62.9% of the respondents said that they did not currently perform FLACS but may in the future, while 26.7% answered that they did not perform it and did not intend to. Accordingly, the proportion of respondents who did not perform FLACS was 89.6%. Of the remaining respondents, 9.4% said that they perform FLACS and will continue to do so, while 1.0% reported that they had performed FLACS but will not continue to do so. In short, it seemed that 10.4% of the respondents had the opportunity to use this advanced technology. It is also possible that there is a group who does not use this technology even though they have access to it.

### Use of Available Technology

Questions to reflect the extent to which doctors apply their knowledge and experience while using available technologies were evaluated in two subgroups, those related to preoperative preparation and those related to methods used during the surgery.

**Figure 1:**
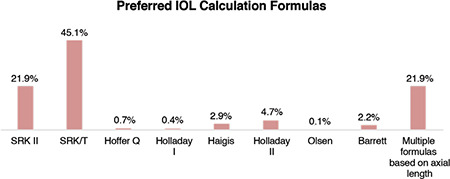
Participants’ preferred formulas for intraocular lens (IOL) power calculation

We examined the responses regarding the IOL calculation formulas used during preoperative preparation. The most commonly used formula was SRK-T, at 45.1%. This was followed by the use of multiple formulas according to axial length (21.9%) and the latest generation formulas Holladay-2 (4.6%), Olsen (0.1%), and Barrett (2.2%) (Figure 1).

The distributions of the respondents’ preferred incision size and location, which are indicators of surgical technique, are shown in Table 3.

The rates of premium IOL usage in cataract surgery reported by Turkish eye surgeons are shown in Table 4. Overall, the mean rates of toric IOL and presbyopia-correcting IOL use were 2.75% and 3.90% of all procedures performed, respectively.

When the 55.7% of the participants who never performed toric IOL surgery were asked why not, the leading reason was inability to acquire them in the hospital where they work (61.4%). The second most common reason was high cost (32.2%). Other deterrents listed by the participants were concern about the lenses’ potential unfavorable objective results (low vision, lens rotation after surgery) (23.6%) and feeling that they would not be financially compensated for their efforts (19.8%).

When the 50.7% of surgeons who never performed presbyopia correction in cataract surgery were asked why they did not, the leading reason cited was high cost (51.4%). The second most common reason cited was concern about the lenses’ potential unfavorable subjective results (halo, glare, poor night vision, patient dissatisfaction) (46.5%). These were followed by concern about the lenses’ potential unfavorable objective results (low vision, low contrast sensitivity) (33.5%) and feeling that they would not be financially compensated (22.1%).

The proportion of respondents who perform intracameral antibiotic injection at the end of the procedure was 89.4% (643 of 719 respondents) and their preferences were using a licensed, ready-to-use cefuroxime product (Aprokam, Thea Laboratories) (42.2%), preparing the injection themselves in the operating room using a cefuroxime vial (31.5%), and preparing the injection themselves in the operating room using moxifloxacin drops (Vigamox, Alcon Laboratories) (26.2%).

## Discussion

The data form this survey were analyzed based on the following inquiries to determine where Turkish ophthalmologists stand in cataract surgey practice: 1) Equipment owned or available and its use in preparation for and during cataract surgery, 2) Whether equipment is used in accordance with current data acquisition criteria, 3) To what extent the preferred surgical techniques and practices serve the purpose of refractive cataract surgery, 4) Use of premium IOL technology, and 5) Sensitivity to the safety of cataract surgery.

### Available Equipment

It was determined that 77.7% of the survey participants were able to use optical biometers (IOLMaster, Lenstar, ALScan, Aladdin, etc.) for IOL power calculation and 67.3% for keratometric measurements. With accuracy to approximately 0.1 mm, ultrasound biometers were widely used in the 1990s; however, toward the end of the 1990s, optical biometers (based on the principle of partial coherence interferometry) reduced the precision to 0.05 mm and became the gold standard.^8^ Based on these data, we can saythat Turkish ophthalmologists have access to these devices, which currently provide the most sensitive and reproducible measurements,^9,10^ and the rate of optical biometry use in a 2017 survey by the European Society of Cataract and Refractive Surgeons (ESCRS) was lower, at 67.1%.^11^ As seen in Table 5, the use of optical biometry for IOL calculation and keratometric evaluations was lower only in secondary public hospitals (23.3% and 17.5%, respectively) and was very high in the 5 other institution subgroups (at least 87% and 74%, respectively) (p<0.001).

Given the contoversy in the literature regarding FLACS in terms of its high cost and its superiority over manual phacoemulsification, it may not be considered correct to regard FLACS as a measure of development in cataract surgery.^12,13^ However, the participants’ responses to the question about FLACS may be meaningful in terms of gaining insight about the medical equipment and advanced technology that the participants have and can use. Our results indicated that 10.4% of the participants are able to use this technique. It is also possible that there is a group that has access to this technology but does not prefer to use it. In a 2017 survey conducted by the American Society of Cataract and Refractive Surgeons (ASCRS), 38% of surgeons performed FLACS.^6^ In this survey, 18% of the respondents practicing in the USA stated that FLACS procedures accounted for more than 21% of their total cataract surgeries. It can be seen that although this technology is used less in Turkey, it still has a considerable presence.

### Use of Equipment in Accordance with Current Data Acquisition Criteria

**Figure 2:**
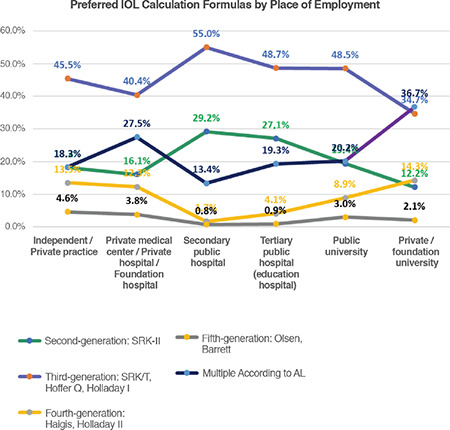
Distribution of preferred IOL power calculation formulas according to the participants’ places of employment AL: Axial length, IOL: Intraocular lens

Preoperative preparation is one of the most important steps affecting postoperative success and patient satisfaction. When the causes of dissatisfaction with refractive cataract surgery are investigated, ametropia is the first and most important reason.^14^ Therefore, in our study, we accepted whether or not a practitioner followed the evolution in IOL calculation formulas as an indicator of development. The introduction of the SRK and SRK-2 formulas in the 1980s can be regarded as the start of IOL power calculation formulas. These experimental data-based regression formulas were replaced by the SRK-T formula in the 1990s. Since then, new theoretical (mathematical) formulas based on geometric optics came into use, followed by more data-based modern formulas that determine or predict effective lens position.^8^ While the success rate in terms of achieving emmetropic outcomes (±0.50 D) ranged from 55-80% with early formulas, it has approached or exceeded 90% with the new formulas.^3^ The Holladay-1, SRK-T, and Hoffer-Q formulas use only 2 variables, while Haigis-3 uses, Barrett-5 uses, and Holladay-2 uses 7 variables. Moreover, there are also now formulas that use artificial intelligence or ray-tracing, which can also now be used together with optical biometers (e.g., Barrett, Olsen, Hill-RBF). It is surprising to see that despite the availability of high-technology equipment, a total of 21.9% of Turkish ophthalmologists are still using SRK-2, a second-generation regression formula. This rate was slightly higher (29.2%) in secondary public hospitals. The most commonly used formulas were SRK-T and other third-generation formulas, which had a reasonable average usage rate of 46.2%, whereas fourth-generation formulas were used by 7.6% and fifth-generation formulas by 2.3% of the participants. In addition, 21.9% of the participants used different formulas according to axial length. The distribution of the participants’ preferred formulas according to the institutions in which they work is shown in Figure 2.

In the ESCRS 2017 survey,^7^ the respondents’ preferences were as follows: SRK-T 75%, Haigis 27%, Hoffer Q 20%, Barrett 18%, Holladay-2 17%, Holladay I 11%, and Olsen 4% (multiple formulas could be selected). When these rates are compared, it can be seen that the ESCRS members use current formulas more.

### Surgical techniques and practices for refractive cataract surgery:

The techniques used during surgery were evaluated to identify the participants’ approaches to eliminate corneal astigmatism and their sensitivity about minimizing SIA.

As cataract surgery has advanced over the last 20 years, there has been a clear trend toward smaller main incisions. In addition to benefits such as faster recovery and visual rehabilitation, less inflammation, and lower risk of endophthalmitis, we know that this improves results by leading to less SIA and astigmatism. It is especially critical for toric and presbyopic correction IOL surgeries to result in less than 0.75 D of corneal astigmatism.^15^

The main cause of SIA is the main cataract incision. Today, incisions 2.2 mm or smaller are considered nearly neutral in terms of astigmatism. However, at incision sizes of 2.8 mm and larger, the likelihood of SIA greater than 0.75 D is significantly higher.^4,16,17^ In the present study, 18.8% of the surgeons used incisions of 2.2 mm or smaller, in accordance with this favorable approach. SIA is acceptable with incisions between 2.3 and 2.7 mm in size, especially when the corneal steep axis is the preferred location. Our results showed that 26.6% of the participants prefer this approach, which is acceptable in terms of SIA. Incisions larger than 2.8 mm can cause SIA that may adversely affect the surgical outcome, and this approach was preferred by 54.6% of the participants.

The only method for reducing existing corneal astigmatism is to place the main incision on the steep corneal axis.^18^ A temporal incision, which is widely preferred, actually has the least effect on the cornea, nearly preserving its present state.^19^ If the surgeon uses a habitual entry site regardless of the preoperative state of the cornea, the existing corneal steep axis may become even steeper or flatter, depending on the situation, in which case the surgeon has no decisive role. Questioning about incision location, which demonstrates the surgeon’s sensitivity regarding corneal astigmatism, showed that 28.9% of the participants tended to make incisions at the corneal steep axis or the nearest possible location, while 26.2% preferred a temporal incision or in the next closest quadrant, which is at least a neutral approach that causes no change in the cornea. Unfortunately, the rate of participants using insensitive approaches, including those that can increase corneal astigmatism, is still very high at 44.9% (Table 3). This negative choice has the potential to reduce the success rates of premium IOL procedures performed by almost half of the participants and is an issue that should be emphasized in terms of education.

### Use of Premium IOL Technology

### Toric IOLs

Rates of toric IOL use are considered an important indicator in terms of following and implementing technical and technological developments in cataract surgery. The literature data show that 1 in every 3 cataract patients has significant astigmatism (≥0.75 D) that affects quality of vision.^20^ In this case, it is normal to expect at least one third of all cataract surgeries to be performed with toric IOLs in order to achieve the goal of refractive cataract surgery. However, only 1.7% of the respondents reported using an approach consistent with this aim (using toric IOLs in at least 21% of cataract surgeries). Although this deficiency is also seen in ASCRS and ESCRS data, it is more apparent in Turkish ophthalmology. According to the ASCRS 2017 survey, 80% of the participants used toric IOLs and 11% used toric IOLs at a rate of over 20%.^21^ In the ESCRS 2017 survey, participants used toric IOLs in 7% of cataract surgeries.^11^ However, this rate was 2.75% in our survey. Another problem was that 55% of all participants did not use toric lenses at all, while this rate was 20% in the ASCRS survey.^21^ Therefore, the clear inadequacy in the area of toric IOL use is noteworthy.

When the participants who did not use toric IOLs (55.7%) were asked why not, non-physician barriers such as difficulty obtaining them (61.4%) and high cost (32.2%) were the leading reasons. Another significant concern was the objective side effects that may occur due to toric lenses (23.6%). Of the 20% of participants in the ASCRS 2017 survey who did not perform toric IOL surgery, high cost was reported as a problem by 49%, and the second most common reason was unavailability, which was reported by 23% of the participants.^21^

The distribution of the 55.7% of Turkish participants who do not implant toric IOLs in 3 of the 4 subgroupings may provide insight into why the usage rate is lower compared to ASCRS members. In terms of professional status, 84.6% of residents, 60.4% of specialists, and 39.1% of faculty members; in terms of academic study, 25.9% of active SCRS members and 62.0% of those who were not active SCRS members; and in terms of age, 70.0% of the 31-40 group and 32.1% of the 51-60 group did not implant toric IOLs. These results show that more advanced career, involvement in academic work in the area of cataract surgery, and older age are associated with significantly higher rates of toric IOL use (p<0.005). Based on these data, we believe that the lack of advancement in toric IOL surgery can be explained by inadequate gains in knowledge and experience, or in other words, lack of education.

### Presbyopia-Correcting IOLs

Presbyopia is being increasingly recognized as an important public health problem. With the global increase in average age, there is now a larger presbyopic population. Presbyopia was estimated to affect about 1.4 billion people in 2000 (23% of the world population), while in 2015 this figure rose to 1.8 billion (25% of the world population). By 2030, the expected presbyopic population is expected to reach 2.1 billion.^22^

At present, the most successful method for the treatment of presbyopia is to implant presbyopia-correcting IOLs during cataract surgery.^14,23^ Looking at the approaches of Turkish surgeons on this subject, we see that 13.7% have reached a rate of 11% of all cataract surgeries or higher. The rate of presbyopia-correcting IOL use in cataract surgeries was 3.9% in our survey, 8% in the 2017 ASCRS survey, and 6% in the 2017 ESCRS survey.^24,25^ In the ASCRS survey, 28% of surgeons did not use them at all.^24^ This rate was higher in our survey, at 50.7%. The reasons cited by Turkish doctors for not using these lenses were high cost (51%), concern about subjective complaints such as poor night vision and dissatisfaction (46%), and concern about potential objective problems such as low vision and loss of contrast sensitivity (33%). Reasons cited by ASCRS members who did not use them were cost (55%), concern about poor night vision (36%), and lack of confidence in the available technology (33%).^24^ ESCRS members attributed not implanting presbyopia-correcting IOLs to cost (60%), the possibility of night vision problems (48%), and concern about loss of contrast sensitivity (40%).^25^ These results show that the barriers to use among both American and European doctors are nearly the same or very similar to those of Turkish doctors. When there is a problem, it occurs all over the world at similar or almost identical rates. But if the problems or excuses are the same, why do Turkish doctors utilize these technologies less? While there was no statistical difference in the barriers to implementation cited in the subgroups, the proportion of respondents not using these lenses at all was 80.6% among residents, 54.0% among specialists, 36.9% among faculty members, 23.0% among active SCRS members, 56.7% among non-SCRS members, 69.0% among those aged 31-40 years, and 24.4% among those 51-60 years of age. The differences were statistically significant (p<0.005). Stated more clearly, the usage rate increases significantly with knowledge and experience. Thus, we can underline that the inadequate results arise from a lack of knowledge and experience i.e., the issue of education as seen with toric IOLs.

### Sensitivity About Cataract Surgery Safety

Participants’ sensitivity regarding the safety of cataract surgery in terms of endophthalmitis can be considered an important measure of development, because numerous studies have demonstrated that this practice reduces the risk of endophthalmitis approximately 5 fold and it has become the gold standard.^26^ In the 2014 ASCRS endophthalmic prophylaxis survey, the rate of intracameral antibiotic use was 50%, and in the 2014 ESCRS survey the rate was 74%.^27,28^ It is highly positive that the frequency of this practice among Turkish ophthalmologists has reached 89.4%, significantly exceeding the figures in Europe and America. However, the rate of using approved products for intraocular injection is still at an intermediate level, at 42.2%.

### Evaluation of the Adequacy of the Participants’ Approaches for Achieving the Objectives of Refractive Cataract Surgery

In the second part of the discussion, we aimed to determine to what extent the participants act in accordance with the objectives of refractive cataract surgery. Table 2 shows the criteria for classifying the participants’ preoperative and surgical approaches as inadequate, standard, and contemporary, according to the literature data^3,4,5^ and the results of recent large international surveys.^6,7^ For this evaluation, we used 5 questions that could correspond to the above classification. Inadequate approach describes the rates of practices that are lacking in terms of achieving the objectives of refractive cataract surgery goals, standard approach reflects the rates of imperfect but acceptable practices, and contemporary approach demonstrates rates of current practices that are ideal for refractive cataract surgery.

Table 6 through 9 present the ratios and statistical comparisons of these 3 approaches among the participant subgroups. In this way, we have attempted to reveal the causes or sources of the inadequate practices discussed above.

Table 6 examines the responses to the 5 questions according to the participants’ professional status and shows that contemporary approaches are generally used significantly more by faculty members, less by specialists, and least by residents. This is another indication that contemporary practices increase with experience and knowledge.

Table 7 shows the comparison according to the institutions where the participants are employed. It can be observed that contemporary approaches are significantly more common in independent/private practice, private medical centers, private/foundation hospitals, and private/foundation universities, whereas inadequate approaches are mostly practiced in secondary public hospitals, followed by tertiary (education) public hospitals and public universities. These results suggest that the working environment of the private sector is more suitable or stimulating for contemporary practices, and that experienced and knowledgeable doctors may work in these environments more frequently.

We believed that participants’ involvement in academic studies in the area of cataract may be an important factor because their knowledge would affect the results. For this reason, active membership in TOA-SCRS was evaluated and responses to the 5 relevant questions were also evaluated according to this criterion. The results in Table 8 show that active members had higher rates of contemporary approaches for all questions when compared to participants who were not active members, and the differences were highly statistically significant.

Table 9 presents the evaluation according to age group. As the age variable is expected to correlate with knowledge, this criterion can also be considered valuable for the purpose of determining rates of insufficient or contemporary approaches. Considering that education and training are still ongoing for the 20-30 group and that participants over 60 may be retired or less active, it could be more meaningful to exclude these groups and compare the 31-40 and 51-60 age groups. When these two groups were compared statistically, we observed highly significant differences for each question, with the rate of contemporary approaches increasing substantially with age.

## Conclusion

In summary, we can state that a large proportion of TOA members have access to technological equipment for preoperative preparation and operations related to cataract surgery, but do not adequately use them to achieve the goals of refractive cataract surgery. Lack of experience and knowledge play a role in this shortcoming, and current approaches regarded as more advanced are seen in areas of specialized research and education.

## Figures and Tables

**Table 1:**
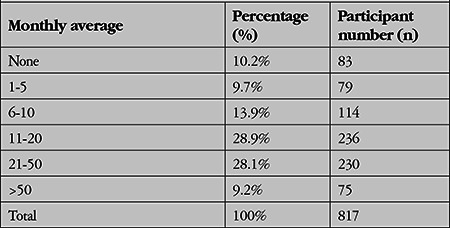
Average monthly cataract surgery volume reported by the participants

**Table 2:**
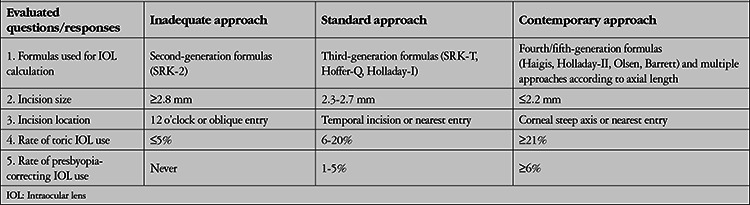
Questions and criteria used to evaluate the level of development of the participants’ cataract surgery practices

**Table 3:**
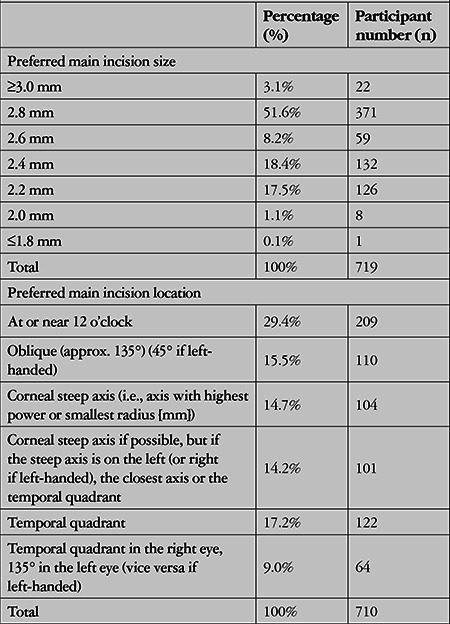
Participants’ preferences for main incision size and location in cataract surgery

**Table 4:**
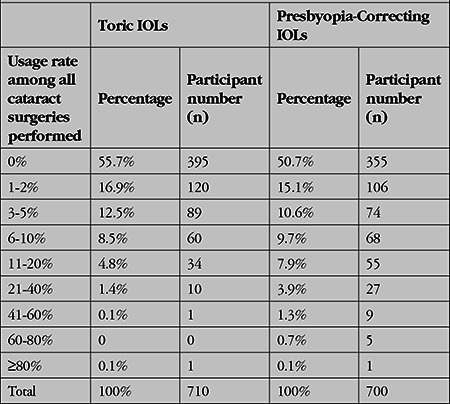
Participants’ toric and presbyopia-correcting intraocular lens (IOL) usage rates as proportion of total cataract surgeries

**Table 5:**
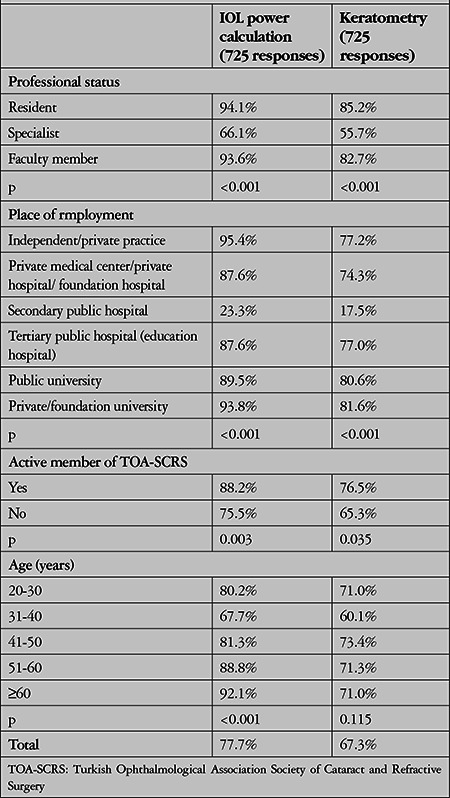
Rates of optical biometry use in intraocular lens (IOL) power calculation and keratometric measurement

**Table 6:**
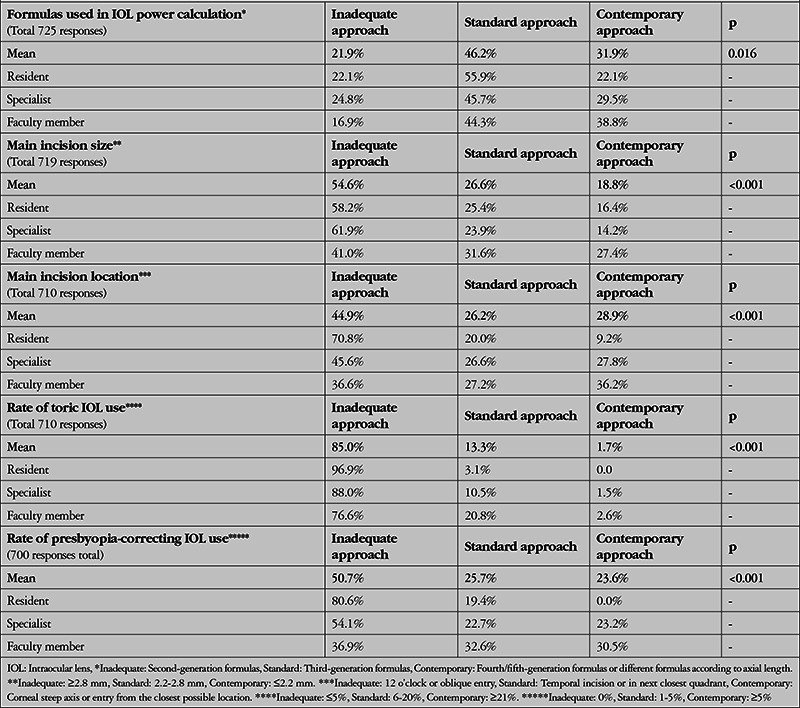
Evaluation of the participants’ level of practice development according to professional status

**Table 7:**
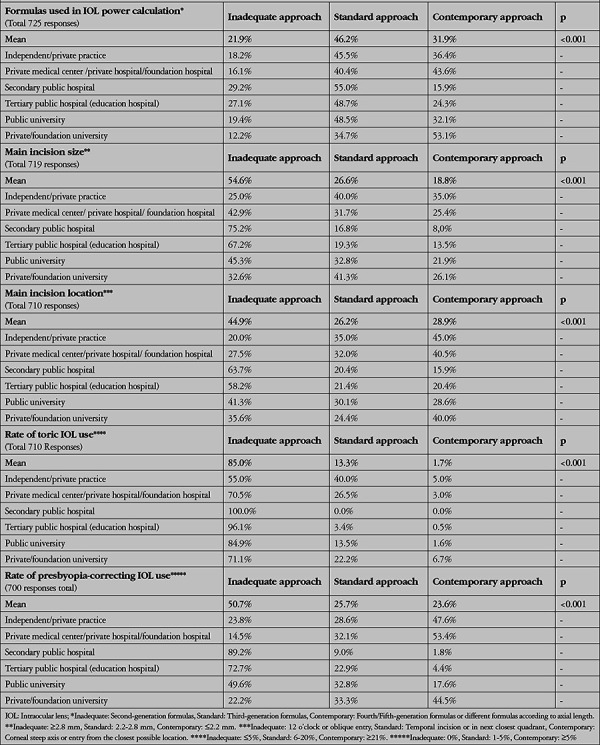
Evaluation of the participants’ level of practice development according to place of employment

**Table 8:**
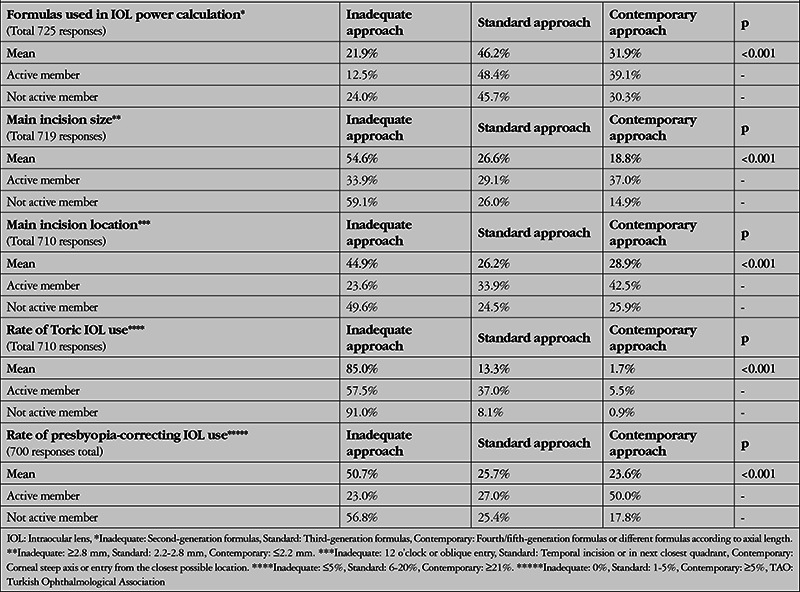
Evaluation of the participants’ level of practice development according to active membership in the TOA Society of Cataract and Refractive Surgery

**Table 9:**
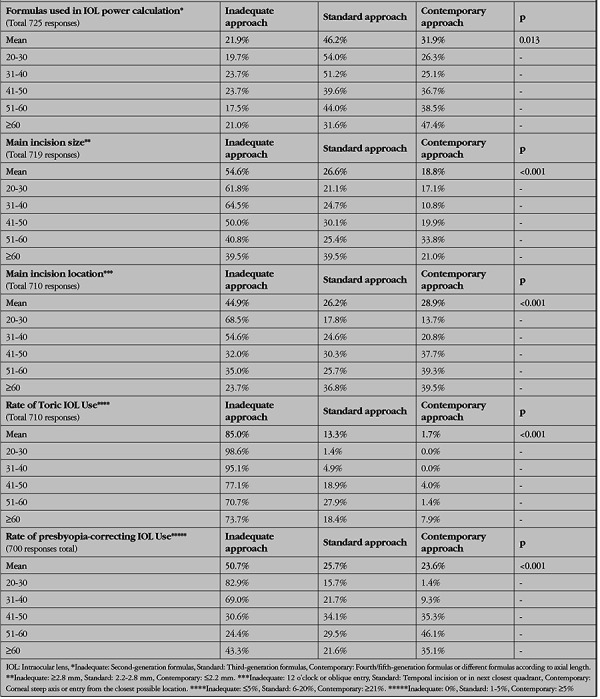
Evaluation of the participants’ level of practice development by age group
